# An Audit on Driving Advice After Hospitalization in a Mental Health Unit

**DOI:** 10.1192/bjo.2022.436

**Published:** 2022-06-20

**Authors:** Mohammed Elsankary, Israa Fawaz, Farhan Shazad, Maged Elashmawy

**Affiliations:** 1 Southern Health NHS Foundation Trust, Basingstoke, United Kingdom; 2 North Hampshire Hospital, Basingstoke, United Kingdom

## Abstract

**Aims:**

To ensure driving status is confirmed on admission (Target 100%) and to confirm driving advice is given to all patients deemed unfit to drive (Target 100%) and to ensure adequate documentation is made in online clinical notes with regards to discussions about driving

**Methods:**

The first cycle of data involved collecting retrospective data from two acute adult psychiatric units and one old age mental health ward. The first cycle of data consisted of inpatients admitted over a two month period in 2020 (36). Data were collected from OpenRio progress notes, OpenRio ward round notes and patient discharge summaries. Following the implementation of interventions the second cycle of data were collected over a 2 month period in 2021. 51 patients met the inclusion criteria for this.

**Results:**

Following our interventions, 47% (24) of patients had their driving status confirmed on/during admission compared to 42% (15) in the first cycle. 15 current drivers were identified in the second cycle.

Of the confirmed drivers, there was a 6% improvement of patients informed they were unfit to drive. A 22% increase in patients given DVLA driving advice was also noted. DVLA notifications increased by 18% following the interventions.

**Conclusion:**

This quality improvement project has shown that educational awareness through teaching sessions and written guidance can improve adherence to national legal guidance. However, further work is required to ensure all psychiatric patients receive adequate information regarding their fitness to drive.

